# Yellow Fever Outbreak, Southern Sudan, 2003

**DOI:** 10.3201/eid1009.030727

**Published:** 2004-09

**Authors:** Clayton O. Onyango, Antoinette A. Grobbelaar, Georgina V.F. Gibson, Rosemary C. Sang, Abdourahmane Sow, Robert Swanepoel, Felicity J. Burt

**Affiliations:** * World Health Organization Collaborating Centre for Arbovirus and Viral Hemorrhagic Fever Reference and Research at Kenya Medical Research Institute, Nairobi, Kenya;; † National Institute for Communicable Diseases, Sandringham, Johannesburg, South Africa;; ‡ World Health Organization of Southern Sudan, Nairobi, Kenya

**Keywords:** genetic characterization, outbreak, yellow fever virus, dispatch

## Abstract

In May 2003, an outbreak of fatal hemorrhagic fever, caused by yellow fever virus, occurred in southern Sudan. Phylogenetic analysis showed that the virus belonged to the East African genotype, which supports the contention that yellow fever is endemic in East Africa with the potential to cause large outbreaks in humans.

Yellow fever virus (genus *Flavivirus*, family *Flaviviridae*) is a mosquitoborne virus endemic in tropical regions of Africa and South America, where it has been responsible for large epidemics. The virus is maintained in sylvatic transmission cycles involving nonhuman primates but uses humans as the sole vertebrate host in urban epidemics. In South America, the sylvatic vectors belong to the *Haemogogus* and *Sabethes* genera, and the urban vector is *Aedes aegypti*; in Africa, *Aedes* species mosquitoes serve as both sylvatic and urban vectors. The virus causes febrile disease with necrotic hepatitis in humans, and death rates can exceed 50%. Although an effective vaccine is available, the virus remains a major public health threat, particularly in Africa, where vaccination is limited by poverty, civil wars, and the inaccessibility of rural areas where outbreaks of the disease occur. The World Health Organization estimates 200,000 cases of the disease occur annually and 30,000 deaths, but because of underreporting, only a small percentage of these cases are recorded.

The first genetic studies of yellow fever virus identified three topotypes, which corresponded with the virus's geographic distribution in West Africa, Central and East Africa, and South America (*1*). In a more recent study, which included a larger number of isolates, greater diversity was identified in Africa, with five genotypes designated: Central/East Africa, East Africa, Angola, West Africa I, and West Africa II (*2*). We report the genetic characterization of yellow fever virus isolates from an outbreak of the disease in southern Sudan in 2003.

## The Study

During the first week of May 2003, the Early Warning and Response Network, established in 1999 in southern Sudan, reported an outbreak of fatal hemorrhagic fever of unknown etiology in the Imatong region of Torit County, which is near the Ugandan border, in a mountainous area covered with tropical rain forest. During the civil unrest in early 2002, many residents were relocated to an internally displaced persons camp in Ikotos County, but in 2003, a number of the residents moved back to the Imatong region. During April and May 2003 suspected cases of hemorrhagic illness were reported, and blood samples collected from Sarianga, Itohom, Lenyleny, Tarafafa, Lofi, and Locomo villages were tested at the Kenya Medical Research Institute (KEMRI), in Nairobi, where yellow fever virus was identified as the causative agent of the outbreak. Aliquots of specimens were submitted to the Special Pathogens Unit at the National Institute for Communicable Diseases in Johannesburg for confirmation of the diagnosis. A report dated June 19, 2003, from the Office of the United Nations Resident and Humanitarian Coordinator for the Sudan described a steady decline in the number of cases occurring during the 4 weeks before the report with a total of 162 suspected cases with 48 deaths in Torit County. However, the report also noted that the recording of suspected cases was inadequate, the figures quoted were an estimate, and final case numbers and deaths were uncertain. The details of the outbreak and laboratory investigations are reported in a separate publication.

Virus isolates were obtained from three patients, two from Locomo village and one from Lofi village. The viral isolations were performed by injecting serum samples from all the patients into suckling mice. RNA was extracted from harvested mouse brain tissue by using Trizol reagent (Gibco BRL, Life Technologies, Gaithersburg, MD), according to the manufacturer's directions. A 670-bp region of the polyprotein gene, which included the 3´ end of the premembrane protein gene, the complete membrane protein gene, and the 5´ end of the envelope protein gene, was amplified by using reverse transcription–polymerase chain reaction (RT-PCR) (*2*). The nucleotide sequence for each amplicon was determined by using Big Dye Terminator Sequencing Ready Reaction kits with AmpliTaq DNA polymerase FS, as described by the supplier (Applied Biosystems, Warrington, UK). A consensus sequence was established by aligning the sequences obtained from the Sudan 2003 outbreak of yellow fever with sequences obtained from GenBank for yellow fever isolates from previous outbreaks of the virus in Africa (Table). The sequences from GenBank were selected to represent the five distinct genotypes circulating in Africa (*2*).

**Table:** History of yellow fever isolates included in the study

Strain	Origin	Year	Source	GenBank no.
SPU160/03/23	Sudan	2003	Human	AY690831
SPU160/03/24	Sudan	2003	Human	AY690832
SPU160/03/25	Sudan	2003	Human	AY690833
14FA	Angola	1971	Human	AF369669
HD 38564	Burkina Faso	1983	Human	AF369670
Serie 227	Ethiopia	1961	Human	AF369674
85-82H	Ivory Coast	1982	Human	U54798
BC 7914	Kenya	1993	Human	AF369676
IB AR 45244	Nigeria	1969	Mosquito	AF369677
H117491	Nigeria	1987	Human	AF369682
Rendu	Senegal	1953	Human	U89338
M 90-5	Sudan	1940	Human	AF369692
M 112-4	Sudan	1940	Human	AF369693
A 709-4-A2	Uganda	1948	Human	AF369694
MR 896	Uganda	1948	Human	U52422
SE 7445	Uganda	1964	Human	AF369695
LSF 4	Zaire	1958	Human	AF369697
SH1464	Senegal	1965	Human	AF369688

Alignment of the nucleotide sequence data was performed by using DNASIS for Windows version 2.5 (Hitachi Software Engineering America, San Francisco, CA). The phylogenetic analysis was performed on a 572-bp region of the amplicons by using a weighted maximum parsimony method, transversion:transition weighting of 5:1, and Phylogenetic Analysis Using Parsimony (PAUP) software version 4.0b4a for Macintosh (*3*). Bootstrap confidence intervals were calculated from 100 heuristic search replicates. The tree (Figure) shows that the virus circulating during the recent outbreak in southern Sudan was closely related to an isolate from an outbreak in Kenya in 1993 that belonged to the East African genotype. Sequence divergence was determined by using Molecular Evolutionary Genetics Analysis (MEGA) version 2.1 (*4*) to calculate the average p-distances or the proportion of pairwise differences within and between groups. The recent Sudan isolates clustered with the East African isolates. A pairwise comparison of nucleotide sequences within the group showed an average distance of 0.111 and 0.179 between the Sudan isolates and an isolate from Kenya (1993) and isolates from Uganda (1948), respectively. No insertions or deletions were found in the Sudan nucleotide sequences compared with the reference strains; however, a number of nucleotide substitutions occurred, most of which were synonymous, and a pairwise comparison of the predicted amino acid sequences showed a high degree of homology. The predicted amino acid sequences were identical for the Kenyan and the Sudan isolates, while the p-distance calculated between the Sudan and Ugandan isolates from 1948 was 0.016. The nucleotide heterogeneity between isolates from East and Central Africa (1.6%–11.6%) supports the concept that different genotypes are circulating and that outbreaks are not the result of imported virus from urban areas (*2*).

**Figure:**
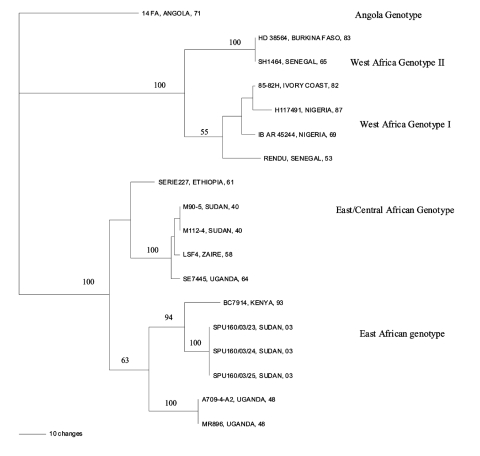
Phylogenetic tree showing the relationship between yellow fever virus circulating during the outbreak in southern Sudan in 2003 and other isolates from previous outbreaks in Africa, determined by using a 572-bp region of the genome, a weighted parsimony method and Phylogenetic Analysis Using Parsimony (PAUP) software. Node values indicate bootstrap confidence values generated from 100 replicates (heuristic search).

## Conclusions

Outbreaks of yellow fever virus have frequently been reported from areas within West Africa since the 18th century, with far fewer outbreaks being identified in East Africa. Serologic evidence for the presence of yellow fever virus in Sudan, Kenya, Uganda, and Tanzania was first reported in 1936 (*5*). However, not until 1940 was the first epidemic confirmed in East Africa, in central Sudan (*5*). Sporadic cases were identified annually in East Africa until 1959, when an outbreak was recorded in the Blue Nile region of Sudan and subsequently in the neighboring region of Ethiopia (*6*). From 1960 to 1962, the largest outbreak to date in Africa occurred in southwest Ethiopia. Additional serologic studies confirmed that yellow fever activity was widespread in Uganda, Somalia, Ethiopia, and Kenya (*6*). Although two possible cases of yellow fever in Kenya were reported in 1943 (*7*), not until 1992–1993 was a large outbreak confirmed in the Rift Valley province of Kenya (*7**,**8*). Subsequent sporadic isolations of virus have been made in East Africa since then, but no large epidemics were recognized until the outbreak in southern Sudan in 2003. Originally, researchers speculated that outbreaks in East Africa were less frequent and smaller than the large outbreaks recorded in West Africa because they were the result of virus's being introduced into the area at the time of the outbreak. However, the genetic data suggest that yellow fever virus is endemic in East and Central Africa, with outbreaks occurring as a result of favorable environmental conditions (*2*). The fact that the isolates from Sudan were closely related to an isolate obtained 10 years ago in Kenya supports the contention that yellow fever is endemic in East Africa and has the potential to cause large outbreaks when conditions favor transmission to humans.
